# Fungal contamination, mycotoxins, histamine, and allergen-related immunoreactivity in edible insects and insect-fortified complementary foods

**DOI:** 10.3389/ffunb.2026.1798430

**Published:** 2026-09-14

**Authors:** Maryam Imbumi, Sheila Okoth, Kris Audenaert, Hans De Steur, Xavier Gellynck, Chrysantus Mbi Tanga, Sarah De Saeger, Judith Kimiywe, Katleen Raes

**Affiliations:** 1 Research Unit VEG-i-TEC, Department of Food Technology, Safety and Health, Ghent University, Kortrijk, Belgium; 2 Division of Agri-Food Marketing and Chain Management, Department of Agricultural Economics, Ghent University, Gent, Belgium; 3 Department of Biology, University of Nairobi, Nairobi, Kenya; 4 Laboratory of Applied Mycology and Phenomics, Department of Plants and Crops, Ghent University, Gent, Belgium; 5 Insects for Food, Feed and Other Uses (INSEFF) Programme, International Centre of Insect Physiology and Ecology (icipe), Nairobi, Kenya; 6 Department of Bioanalysis, Centre of Excellence in Mycotoxicology & Public Health, Campus Heymans, Faculty of Pharmaceutical Sciences, Ghent, Belgium; 7 Department of Food, Nutrition and Dietetics, Kenyatta University, Nairobi, Kenya

**Keywords:** allergen-related immunoreactivity, edible insects, fungal contamination, histamine, insect-fortified complementary foods, mycotoxins, storage

## Abstract

**Introduction:**

Edible insects are consumed by over 3,000 ethnic groups, with 2,205 species documented as food in 128 countries, yet evidence on fungal contamination, mycotoxins and allergen-related risks remains limited. This study assessed these hazards in edible insects and insect-fortified complementary foods for infant and young child nutrition.

**Methods:**

Fungal contamination in 11 edible-insect products, seven food ingredients, seven insect-enriched formulations and five commercial complementary flours was assessed by culture and internal transcribed spacer sequencing. Fungal isolation comprised 1,080 inoculated culture observations across three independent batches per material type, from which 798 fungal isolates were recovered. Endpoint PCR assessed aflatoxin- and fumonisin-biosynthesis markers in **Aspergillus* flavus*-like and Fusarium-like morphotypes. Mycotoxins in five complementary-food formulations were quantified by LC–MS/MS over three months, while histamine concentrations and lysozyme- and crustacean-reactive signals were measured in edible-insect preparations by enzyme-linked immunosorbent assays.

**Results:**

*Aspergillus* accounted for 70.7% of isolates from insect products. *Penicillium* and Fusarium were detected in all 11 and eight insect products, respectively. Among 44 A*. flavus*-like morphotypes, *aflD* and *aflQ* were detected in 32 and 23, respectively; among 22 Fusarium-like morphotypes, *VERTF* and *FUM1* were detected in 10 and 19, respectively. Marker detection did not establish gene expression or mycotoxin production. Six of 23 mycotoxins were detected: 3-acetyldeoxynivalenol, aflatoxin G1, sterigmatocystin, and fumonisins B1, B2 and B3. Only aflatoxin G1 and fumonisin B1 were quantified, at 4.20 µg/kg and 20.31–86.88 µg/kg, respectively; all other confirmed detections were below quantification limits. Quantifiable fumonisin B1 occurred at later storage points and peaked after three months, although patterns were not uniformly monotonic across formulations. The aflatoxin G1 point estimate was below the Kenyan total-aflatoxin maximum, and the European Union maize-based infant-food fumonisin value was used only for context; neither comparison established compliance or safety. Quantifiable histamine concentrations ranged from 3,108 to 30,232 µg/kg; three preparations were < 2,500 µg/kg and one was > 40,000 µg/kg. Lysozyme-reactive signals were < 25 µg/kg in all 17 preparations. Crustacean-reactive signals were > 400 µg/kg in 16 preparations, while the remaining result was 382 µg/kg.

**Discussion:**

These findings support fungal-contamination prevention, controlled storage and assessment of potential allergen cross-reactivity in edible insects.

## 1 Introduction

Edible insects are widely consumed across the tropics and subtropics and remain part of the traditional diets of more than 3000 ethnic groups worldwide (Omuse et al., 2024). The most recent global synthesis documented 2205 edible insect species consumed in 128 countries, extending earlier estimates of roughly 1500–2000 species in 113–124 countries (Omuse et al., 2024). In Africa, 464 species have been reported from 48 countries, with the greatest diversity in Central Africa followed by Southern Africa; Kenya falls within the group of countries with 10–50 documented edible insect species, although earlier Kenya-specific sources have reported 17 species used for food or feed (Omuse et al., 2024).

Globally, the most represented edible insect orders are Coleoptera, Hymenoptera, Lepidoptera, and Orthoptera, broadly consistent with earlier summaries in which beetles, caterpillars, wasps-bees-ants, and crickets-grasshoppers-locusts accounted for most recorded edible taxa (Omuse et al., 2024). In Africa, however, Lepidoptera are especially prominent, and termites, although fewer in species number, are likely among the most widely eaten insects by biomass (Omuse et al., 2024). Edible insects provide diverse essential nutrients, including high-quality protein, lipids, vitamins, minerals, and other bioactive components, although composition varies by species, developmental stage, diet, and processing (Omuse et al., 2024).

Edible insects are prepared and processed in multiple ways, for example salted *Ruspolia differens* in East Africa, insect oils (e.g. *Clanis bilineata*) and fermented sauces from *Tenebrio molitor* (Borremans et al., 2018; Mmari et al., 2017). Insect-based bakery products and extruded snacks or instant porridges show good market potential (Azzollini et al., 2018; Imbumi et al., 2024; Osimani et al., 2018).

At the same time, undernutrition was pervasive. In early 2023, 45.8 million people in the Greater Horn of Africa, including 5.4 million in Kenya, were acutely food insecure. Nearly 12 million children younger than 5 years, including about 1 million in Kenya, were acutely malnourished, and almost 3 million, including about 250,000 in Kenya, required treatment for severe acute malnutrition (World Health Organization, 2023). In 2022, 30% of African children under 5 were stunted and 5.8% wasted; in Eastern Africa the figures were 30.6% and 5.0%, and in Kenya 18.4% and 4.9%, respectively (United Nations Children’s Fund, World Health Organization, and World Bank Group, 2023). The acceptability of amaranth-based complementary foods containing edible termites and dagaa fish among young children and their mothers in western Kenya has been demonstrated (Konyole et al., 2012). Insect-fortified complementary foods may therefore contribute to nutrient density and dietary diversity, provided that they are safe, acceptable, affordable and accessible.

Food-safety concerns may constrain the wider use of edible insects and include microbial pathogens, chemical contaminants and allergenic proteins introduced through rearing substrates, harvesting, processing or storage (Gałęcki et al., 2023). Although insects do not synthesize mycotoxins, they may retain or accumulate mycotoxins such as aflatoxin B1, ochratoxin A and fumonisin B1 from contaminated substrates (Mancini et al., 2020). In multi-ingredient complementary foods, grains and other plant ingredients may provide additional sources of fungal and mycotoxin contamination. Temperature, moisture, crop stress and storage conditions influence fungal ecology and mycotoxin formation, and these factors may be altered by climate change (Perrone et al., 2020).

Histamine and allergenic proteins represent distinct additional hazards. Histamine can form through microbial decarboxylation of histidine during inadequate handling or storage, is heat-stable and has caused poisoning outbreaks following insect consumption (Chomchai and Chomchai, 2018; Chumjai et al., 2023). Lysozyme-related proteins occur in insect tissues, whereas hen’s-egg lysozyme is an established food allergen; however, clinically relevant cross-reactivity between egg and insect lysozymes has not been established. More extensively documented arthropod cross-reactivity involves proteins such as tropomyosin and arginine kinase. Some consumers sensitized to crustaceans or house-dust mites may recognize homologous insect proteins, although the clinical relevance and effects of processing vary among proteins, insect species and individuals (De Marchi et al., 2021; Yang et al., 2023; Karnaneedi et al., 2024; Alejandro Ruiz et al., 2025).

Despite increasing interest in insect-fortified foods, limited evidence jointly addresses fungal contamination, storage-associated multi-mycotoxin patterns, histamine and allergen-related immunoreactivity in complementary-food contexts. This study therefore investigated (i) fungal contamination in edible insects, food ingredients, insect-fortified formulations and commercial complementary flours; (ii) the occurrence and concentration patterns of mycotoxins during three months of storage in insect-fortified formulations and a non-insect control; and (iii) histamine concentrations and lysozyme- and crustacean-reactive enzyme-linked immunosorbent assay signals across edible insect species and processing states. The study aimed to characterize safety-relevant contamination and identify priorities for processing, storage and monitoring of insect-derived complementary foods intended for infant and young-child nutrition.

## 2 Materials and methods

### 2.1 Insect sample collection and processing

Eleven edible insects were used in this study, which included: wing termites (*Macrotermes subhyalinus*), lake-flies (*Chaoborus* sp.), crickets (*Gryllus bimaculatus* and *Scapsipedus icipe*), desert locusts (*Schistocerca gregaria*), grasshoppers (*Ruspolia differens*), silkworm pupae (*Bombyx mori*), wild silk moth (*Anaphe panda*), mopane worms (*Gynanisa maja* and *Gonimbrasia belina*), *Bunaea alcinoe* and *Gonimbrasia zambesina*. The desert locusts (Cheseto et al., 2020) and crickets were mass-reared at the Animal Rearing and Containment Unit (ARCU) of the International Centre of Insect Physiology and Ecology *(icipe)*, Nairobi, Kenya. The grasshoppers (*R. differens*) were wild harvested in Nakawa and Nakidye divisions, Uganda, packed in polythene zip-lock bags and transported in cooler boxes at -20 °C. The caterpillars, such as *G. maja* and *G. belina* were obtained from Botswana, while *B. mori*, *A. panda*, *G. zambesina* and *B. alcinoe* were collected in Kenya and processed accordingly (Kusia et al., 2021). The collection, identification, packaging and storage of fresh raw insect samples into plastic zip-lock bags was conducted at *icipe* for further analysis. The insect species were purposively selected because they are commonly consumed in the study regions or were available through *icipe* and regional collaborators. The selection was intended to provide broad exploratory coverage of culturally relevant edible insects rather than a statistically representative sample of all edible-insect species consumed in Africa.

#### 2.1.1 Processing techniques of insect samples

Some wild-collected insects from Kenya, Uganda and Botswana were obtained and processed in bulk lots of approximately 20–50 kg. For each insect species and analytical workflow, separate sample portions were retained and processed without pooling. Portions used for flour-based fungal, mycotoxin and ELISA analyses were dried, milled and sieved, whereas ELISA samples analyzed as fresh/raw, pan-fried or oven-dried preparations were processed separately, as applicable.

The appendages of grasshopper, desert locust and cricket were carefully removed. For each insect species and analytical workflow requiring dried insect powder, a separate 1000 g portion of fresh or frozen insect material stored at -20 °C was washed under tap water and air-dried before being placed in an oven dryer (TD-384KN model, Tokyo Thermo Tec) maintained at 60 °C (Nyangena et al., 2020). The samples were oven-dried to constant weight over 2–3 days, removed from the drying chambers and allowed to cool for 20 min at room temperature (22-25 °C). The dried insects were milled into flour using an electric grinder (MRC laboratory grinder, London, UK) at *icipe* and sieved through a 0.595-mm mesh (Nyangena et al., 2020). Each resulting species-specific insect flour batch (approximately 800 g) was packaged separately in a sterile zip-lock polyethylene bag and stored at −20 °C. Portions of the same flour batch were used for fungal and mycotoxin analyses and, where dried insect powder was required, for ELISA. Raw and pan-fried ELISA preparations were prepared separately as described in Section 2.8.1. No insect materials or flours were pooled across species or analytical workflows.

### 2.2 Acquisition of cereals, grains, tubers and mangoes

This study analyzed contemporary food ingredients from Kenya used to formulate complementary foods. These included the Kenya Agricultural and Livestock Research Organization (KALRO) finger-millet variety U15 (Eleusine coracana) and grain-amaranth seeds (*Amaranthus cruentus* and *Amaranthus hypochondriacus*). Two kilograms of finger millet were obtained from KALRO farmers’ fields based in Mumias, Kenya, while 6 and 2 kg of *A. hypochondriacus* and *A. cruentus*, respectively, were acquired from KALRO farmers’ fields based in Kakamega. Twenty kilograms each of the orange-fleshed sweet-potato varieties KALRO Kabode and Kenspot (Ipomoea batatas) were harvested from KALRO potato fields in Kakamega. Thirty kilograms of medium-sized apple mango variety (*Mangifera indica*) were purchased from Ngara markets, Nairobi, Kenya. Finally, 1 kg of full cream cow milk powder was purchased from Nairobi supermarkets.

#### 2.2.1 Processing of cereals, grains, tubers and mangoes

Finger millet (6 kg) was sorted separately, sun-dried and milled into flour at a regular miller in Mumias, Kenya and then packaged into khaki (sugar) bags. Then amaranth grains (6 kg of *A. hypochondriacus* and 2 kg of *A. cruentus*) were separately sorted, sun-dried and milled into flour at a milling machine in Kakamega, Kenya and packaged in khaki (sugar) bags. These cereals and grain samples were packed in tightly closed boxes, transported to Nairobi by courier services and kept under room temperature for further analysis. For the sweet potatoes, 20 kg of Kenspot and 20 kg of Kabode were washed with tap water, peeled, rinsed again and grated. The grated roots were placed on canvas papers and sun-dried for 3–6 hours. The dried potato chips were packaged in khaki (sugar) bags and milled into flour at a regular miller in Kakamega town. At least 4 kg of each variety of sweet potato flour was separately packed in boxes, transported to Nairobi by courier and refrigerated till analysis. The apple mangoes (30 kg) were washed under running tap water, peeled and destoned. The mangoes were grated into chips, oven-dried at 60 °C and then blended into flour in a kitchen blender to obtain 3 kg mango powder. The mango powder was kept in tightly locked plastic containers and refrigerated till analysis.

### 2.3 Insect-fortified formulations developed

Six selected insects and other ingredients were used to formulate insect-fortified complementary foods as described by Imbumi et al. (2024). Nine formulations were made, five formulations prepared without milk powder, consisting of grain amaranth (*A. hypochondriacus*) and 10% insects such as grasshopper (*R. differens*) (GH+O) or desert locusts (*S. gregaria*) (LOC+O), or lake-flies (*Chaoborus* sp.) (LKF+O), or crickets (*S. icipe*) (CR+O) and dewinged termites (*M. subhyalinus*) (TM+O). Finally, 10% cow milk powder was added to grain amaranth seeds: *A. hypochondriacus* and crickets (*S. icipe*) (CR+M), *R. differens* (GH+M) and dewinged termites (TM+M). The “control” formulation had no insect (M+O) and consisted of enriching the basic ingredients with milk powder to generate a different product. For each formulation included in fungal analysis, 1000 g of flour was prepared. For the mycotoxin shelf-life experiment, a separate 200 g formulation portion was prepared for each bottle by weighing and mixing ingredients obtained from the same ingredient batches. Twelve bottles were prepared for each of five selected formulations, giving 60 tightly sealed plastic bottles with a total capacity of 500 g each. Three bottles per formulation were assigned to each of four sampling periods: baseline and one, two and three months (**Figure 1**). The packaged formulations were stored in tightly closed bottles on laboratory shelves for three months at approximately 22 °C and an estimated ambient relative humidity of 45%. Relative humidity was not actively controlled or continuously monitored.

**Figure 1:**
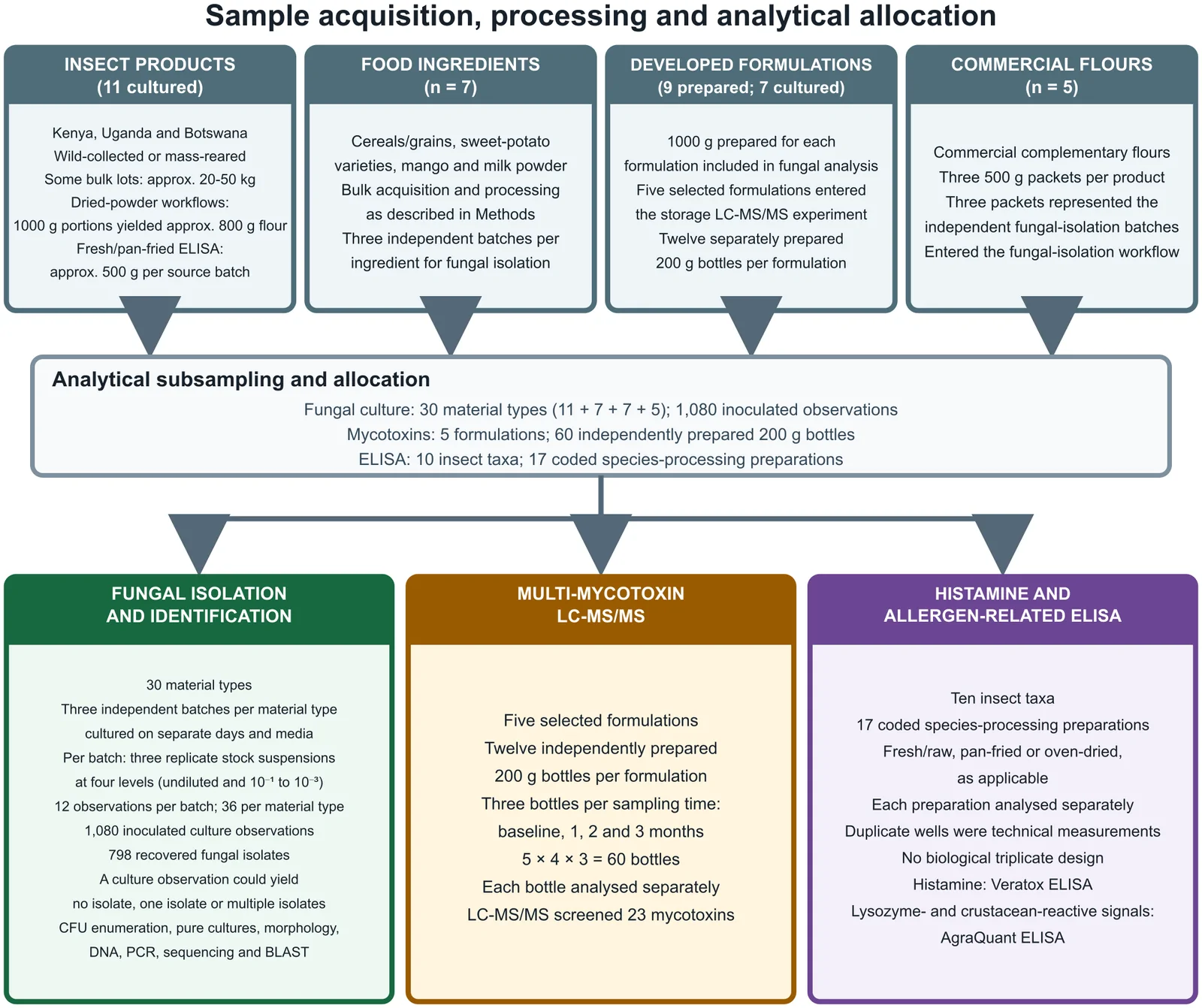
Sample acquisition, processing and allocation to the fungal-isolation, multi-mycotoxin and enzyme-linked immunosorbent assay workflows. Fungal isolation included 11 insect products, seven ingredients, seven developed formulations and five commercial complementary flours. Three independently collected or prepared batches of each material were cultured separately on different days: one batch on half-strength potato dextrose agar, one on full-strength potato dextrose agar and one on Sabouraud dextrose agar. Within each batch, three replicate stock suspensions were analyzed at four dilution levels, giving 12 culture observations per batch and 36 per material type. These nested observations were not additional independent batches. Multi-mycotoxin analysis included five formulations, four storage periods and three independently prepared bottles per formulation and storage period (60 bottles). The ELISA workflow included 17 coded species-processing entries representing 10 insect taxa. ELISA wells were technical measurements, and no biological triplicate design was used for the allergen-related assays. Each assay-specific preparation was analyzed separately, and no insect materials or extracts were pooled.

### 2.4 Control commercial flours

Commercial complementary porridge flours for children formed the second group of formulations that were the “control” purchased for the study. The ingredients in these flours include finger millet, wheat, sorghum, whole maize, refined maize (fermented) flours, grain amaranth seeds, soya beans, groundnuts, and cassava, industrially fermented or soured (with souring agents). These were grouped as commercial flours A (soured) and B from Nairobi, and commercial flours C (fermented), D and E (both soured with souring agents) from Luanda and Khwisero sub-counties. Three 500-g packets of each commercial flour were purchased (**Figure 1**).

### 2.5 Sample preparation and fungal isolation

Fungal isolation was performed on 30 material types: 11 insect products, seven food ingredients, seven developed complementary food formulations and five commercial complementary flours. Analytical portions were taken from the larger source lots or prepared product units described in Sections 2.1–2.4. For each material type, three independently collected or prepared sample batches were analyzed in separate culture runs performed on different days.

For each batch, three 1-g analytical portions were weighed and prepared separately. Each 1-g portion was suspended in 10 mL of sterile distilled water and vortexed thoroughly to obtain a homogeneous stock suspension (Gebremariam et al., 2021). Streptomycin sulfate was added to each culture medium at 250 mg/L to inhibit bacterial growth during fungal isolation (Alfiky, 2022).

Serial dilutions (10^-^¹ to 10^-^³) were prepared aseptically from each of three replicate stock suspensions prepared from each independent batch. The three independent batches of each material were processed on different days and assigned to different culture media: the first batch was cultured on half-strength potato dextrose agar (PDA; 19.5 g/L, HiMedia), the second batch on full-strength PDA (39 g/L, HiMedia), and the third batch on Sabouraud dextrose agar (SDA; TM Media). For each replicate stock suspension, a 1-mL aliquot of the undiluted stock and of each of its three serial dilutions was inoculated onto the medium assigned to that batch. Each independent batch therefore generated 12 culture observations (3 replicate stock suspensions × 4 dilution levels), and each material type generated 36 culture observations (3 independent batches × 12 observations). The replicate stock suspensions and dilution levels were nested within the originating batch and were not treated as additional independent material batches. The culture-observation total included all inoculated batch-replicate-dilution combinations, including plates with no recoverable fungal growth. A culture observation could yield no isolate, one isolate or more than one morphologically distinct isolate.

The plates were incubated at 29 ± 1 °C for 5–7 days in the dark to facilitate fungal growth (Gebremariam et al., 2021). Colony-forming units (CFUs) were recorded from countable plates, and fungal loads were calculated as follows (Equation 1):

(1) $$\log_{10}(\text{CFU}/\text{g})=\log_{10}[(\frac{N}{d\times V})\times 10]$$

where *N* is the mean colony count from the triplicate plates, *d* is the decimal dilution of the plated suspension, *V* is the volume plated in milliliters, and the factor 10 corrects for the initial suspension of 1 g of sample in 10 mL of sterile distilled water (Gebremariam et al., 2021).

After incubation, morphologically distinct fungal colonies were subcultured onto fresh 9-cm PDA plates and incubated at 29 ± 1 °C for 7 days to obtain pure cultures. The purified isolates were stored at 4 °C for subsequent identification (Ullah et al., 2022). Isolates recovered from the three batch-specific culture runs were aggregated at the material level for morphological and molecular identification and statistical analysis.

Each purified colony selected for identification was assigned a numerical isolate code. When more than one morphologically distinct isolate was selected from the same original material sample, the suffixes A, B or C were appended to the shared numerical code. These suffixes distinguish separate fungal isolates and do not denote sequencing replicates.

#### 2.5.1 Morphological identification of fungal isolates

Fungal isolates were first examined for colony morphology, including color, margin, texture, and growth rate on selective media. These macroscopic features helped distinguish genera and, in some cases, species (Jedidi et al., 2017). Microscopic examination involved staining spores and hyphae, most often with lactophenol cotton blue, and observing them under 40× and 100× magnifications. Diagnostic features such as conidiophore structure, vesicle shape, phialide arrangement, and spore morphology were evaluated (Ekwomadu and Mwanza, 2023; Jedidi et al., 2017). Identification was further guided by dichotomous keys and authoritative monographs (Leslie and Summerell, 2006; Pitt and Hocking, 2009; Samson et al., 2007), which provide detailed descriptions for *Aspergillus*, *Fusarium*, and *Penicillium* species (Ekwomadu and Mwanza, 2023; Jedidi et al., 2017).

For *Aspergillus*, colony color, conidiophore and vesicle structure, and conidial ornamentation were important diagnostic traits (Jedidi et al., 2017). In contrast, *Fusarium* identification relied on macroconidia shape, presence of microconidia, and pigmentation (Ekwomadu and Mwanza, 2023). For *Penicillium*, conidiophore branching and phialide arrangement were key identifying features (Jedidi et al., 2017). The relative frequency (RF) of fungal occurrence for each genus or species was calculated using Equation 2:

(2) Relative frequency (%) = (Number of isolates from genus or species/Total isolates recovered) * 100

After incubation, colonies displaying distinct macroscopic and microscopic morphologies were subcultured on fresh PDA to obtain pure cultures. Representative purified isolates from the recorded morphotypes were selected for molecular identification.

### 2.6 DNA extraction from fungal isolates

Fungal DNA was extracted using a silica-based purification approach. Approximately 0.1–0.2 g of fresh mycelium was scraped aseptically from 5–7-day-old PDA or SDA cultures using sterile spatulas. The material was placed into sterile 1.5 mL microcentrifuge tubes and frozen at –80 °C overnight to facilitate cell lysis. Frozen mycelia were then ground using sterile quartz sand and liquid nitrogen to produce a fine powder, a method shown to enhance DNA yield (Chauhan et al., 2022).

Genomic DNA was extracted using the Quick-DNA™ Fungal/Bacterial Miniprep Kit (Zymo Research), following the manufacturer’s instructions. The method employs a combination of mechanical disruption, lysis buffer, and silica-membrane purification to isolate high-purity DNA suitable for downstream applications. DNA concentration and purity were measured with a NanoDrop 2000 spectrophotometer (Thermo Scientific) and stored at –20 °C until PCR analysis (Chauhan et al., 2022).

#### 2.6.1 Molecular identification of fungal isolates

Representative fungal isolates were characterized by PCR amplification of the nuclear ribosomal internal transcribed spacer region (ITS1–5.8S–ITS2) using the primers ITS1F and ITS4 (Schoch et al., 2012). Amplification was performed using primer pair ITS1F (5′-CTTGGTCATTTAGAGGAAGTAA-3′) and ITS4R (5′-TCCTCCGCTTATTGATATGC-3′), as described by Gardes and Bruns (1993), targeting a ~595 bp fragment.

Each 25 μL PCR reaction consisted of: 12.5 μL GoTaq^®^ G2 Hot Start Green Master Mix (Promega), 1 μL of forward primer (10 μM), 1 μL of reverse primer (10 μM), 1 μL of fungal genomic DNA (~20–50 ng), and 9.5 μL of nuclease-free water. Amplification was conducted under the following thermocycling conditions (White et al., 1990):

1. Initial denaturation: 95 °C for 2 minutes,

2. 30 cycles of:

- Denaturation at 95 °C for 30 seconds,
- Annealing at 55 °C for 30 seconds,
- Extension at 72 °C for 1 minute

3. Final extension: 72 °C for 5 minutes.

Amplicons were separated by electrophoresis on a 1.2% agarose gel in 1× TAE buffer and visualized under UV light after staining with ethidium bromide. Successfully amplified products were purified using the Wizard^®^ SV Gel and PCR Clean-Up System (Promega). Initial Sanger sequencing was performed on 344 purified PCR products by LGC Biosearch Technologies, Germany, using the ITS4 sequencing primer. For isolates whose initial reads failed or were insufficient for taxonomic assessment, stored genomic DNA was re-amplified and sequenced bidirectionally by Inqaba Biotechnical Industries Ltd., South Africa. These were repeat analyses of selected isolates and were not counted as additional fungal isolates. Following sequence-quality assessment and replacement of inadequate reads, 280 unique molecular representatives were included in the taxonomic analysis, comprising 273 ITS records and seven LSU D1/D2 records.

Chromatograms were quality-checked, low-quality terminal regions were trimmed, and ambiguous base calls were manually reviewed in CLC Genomics Workbench version 21.05.5. Processed sequence records were queried against the NCBI nucleotide database using BLASTn (Altschul et al., 1990).

Percent identity, query length, the taxonomic agreement among the leading BLASTn matches and the corresponding morphological identification were considered in the final assignment. The ITS results were used to support genus-level identification. Species-level assignments were not made because ITS alone does not reliably distinguish all closely related species within several *Aspergillus, Penicillium and Fusarium* complexes (Schoch et al., 2012; Raja et al., 2017; Samson et al., 2014; Visagie et al., 2014).

For each sequenced isolate, the nearest named species-level match, NCBI reference accession, percentage identity and query-sequence length were recorded. Genus-level assignments were based on the BLASTn results, including sequence identity and query length, agreement among the leading reference matches and consistency with morphological identification. Because sequence quality, length and species-level discriminatory power varied among isolates, taxonomic assignments were reported conservatively at genus level (Schoch et al., 2012; Samson et al., 2014; Visagie et al., 2014; Raja et al., 2017). Where the highest-quality BLASTn matches did not permit unambiguous species-level discrimination, no species-level assignment was made.

**Supplementary Table S3** provides the nearest reference matches and sequence metadata for the molecular representatives included in the taxonomic analysis.

For seven isolates for which usable ITS reads were not obtained, replacement sequence records generated by Inqaba comprised paired reads of the D1/D2 region of nuclear large-subunit ribosomal DNA using primers NL1 (5′-GCATATCAATAAGCGGAGGAAAAG-3′) and NL4 (5′-GGTCCGTGTTTCAAGACGG-3′) (Kurtzman and Robnett, 1998). These LSU D1/D2 sequences were evaluated separately from the ITS sequences and were used to support genus-level identification. The retained ITS and LSU D1/D2 sequences are supplied separately as **Supplementary Data S4** and **S5**, respectively.

#### 2.6.2 Exploratory PCR screening for mycotoxin-associated molecular markers

Following morphological and microscopic classification, selected purified fungal isolates were screened by conventional endpoint polymerase chain reaction (PCR) for DNA markers associated with aflatoxin or fumonisin biosynthetic potential. Forty-four *Aspergillus flavus*-like morphotypes were tested for the aflatoxin-biosynthesis genes *aflD* (*nor-1*) and *aflQ* (*ordA*) using Nor1-F/Nor1-R and Ord1-gF/Ord1-gR, respectively (Okoth et al., 2012). Twenty-two *Fusarium*-like isolates were tested using Fpro-fum1-F/Fpro-fum1-R, targeting the fumonisin-biosynthesis gene *FUM1* (Bluhm et al., 2004), and *VERTF*-1/*VERTF*-2, targeting an intergenic-spacer profile associated with potentially fumonisin-producing *Fusarium verticillioides* strains (Patiño et al., 2004). Expected amplicon sizes were 862 bp for *aflD*, 757 bp for *aflQ*, 183 bp for *FUM1* and 400 bp for *VERTF*. PCR products were evaluated by agarose-gel electrophoresis and recorded as presence or absence of a band of the expected size. Because these endpoint assays targeted genomic DNA, positive amplification indicated detection of the selected marker and did not demonstrate gene expression or mycotoxin production. The isolate-level results of this exploratory marker screening are provided in **Supplementary Table S6**.

### 2.7 Multi-mycotoxin analysis of insect-fortified complementary flours by LC–MS/MS

For the LC-MS/MS mycotoxin storage experiment, five complementary flour formulations were evaluated: GH+O, GH+M, TM+O, TM+M and M+O. These formulations were developed previously and evaluated in an earlier consumer sensory study (Imbumi et al., 2024). The present experiment was an exploratory food-safety assessment of this predefined formulation set. For each formulation, ingredients for 12 separate preparations were weighed and mixed independently using the same ingredient batches. Each preparation was placed in a separate tightly closed plastic bottle. Three bottles per formulation were assigned to each of four sampling times: baseline (fresh/month 0) and one, two and three months of storage. Thus, 60 bottles were prepared in total (five formulations × four sampling times × three independently prepared bottles), giving three independently prepared formulation replicates per formulation at each sampling time (*n* = 3). These replicates were independent at the formulation-preparation and bottle levels but did not represent independent ingredient batches. Ingredients shared among formulations, including grain amaranth, originated from the same respective batches. Bottles assigned to the stored sampling times were placed in cardboard boxes and maintained on laboratory shelves for up to three months at approximately 22 °C. Relative humidity was not actively controlled or continuously monitored; the ambient relative humidity was estimated at approximately 45%. At each sampling time, the three bottles assigned to each formulation were analyzed separately by LC-MS/MS (**Figure 1**).

The analytical procedure followed the laboratory RES-Sorghum protocol, which was based on the multi-analyte sorghum LC–MS/MS method described by Ediage et al. (2015) and developed in accordance with Commission Decision 2002/657/EC and Commission Regulation (EC) No 401/2006. Five complementary porridge formulations previously evaluated in the consumer-acceptance study were analyzed: four insect-fortified formulations and one non-insect control. Analysis was performed using a Waters ACQUITY UPLC system coupled to a Micromass Quattro Premier XE triple-quadrupole mass spectrometer. Data acquisition was performed using MassLynx software, while chromatographic integration, matrix-matched calibration and concentration calculations were conducted using the QuanLynx module. The reported analytical batch comprised 23 target analytes: nivalenol (NIV), aflatoxin B1 (AFB1), aflatoxin B2 (AFB2), aflatoxin G1 (AFG1), aflatoxin G2 (AFG2), ochratoxin A (OTA), deoxynivalenol (DON), zearalenone (ZEN), fumonisin B1 (FB1), fumonisin B2 (FB2), fumonisin B3 (FB3), T-2 toxin (T-2), HT-2 toxin (HT-2), 3-acetyldeoxynivalenol (3-ADON), 15-acetyldeoxynivalenol (15-ADON), diacetoxyscirpenol (DAS), fusarenone-X (FUS-X), neosolaniol (NEO), altenuene (ALT), alternariol (AOH), alternariol monomethyl ether (AME), roquefortine-C (ROQ-C) and sterigmatocystin (STERIG). Chromatographic separation was performed using a Waters^®^ Symmetry C18 analytical column (5 µm, 150 × 2.1 mm) fitted with a Waters^®^ Symmetry C18 guard column (3.5 µm, 10 × 2.1 mm; Waters, Zellik, Belgium). Mobile phase A consisted of water/methanol/acetic acid (94:5:1, v/v/v) containing 5 mM ammonium acetate, while mobile phase B consisted of water/methanol/acetic acid (2:97:1, v/v/v) containing 5 mM ammonium acetate. Chromatography was performed at a flow rate of 0.3 mL/min using the 28-min gradient specified in the RES-Sorghum protocol. The injection solvent consisted of equal volumes of mobile phases A and B.

Matrix-matched calibration used a caterpillar-based complementary food formulation containing approximately 50% grain amaranth, sweet potato flour and other formulation ingredients as the surrogate blank matrix. The blank matrix was fortified at five concentration levels according to the RES-Sorghum protocol. Zearalanone (ZAN) and deepoxy-deoxynivalenol (DOM) were used as internal standards. After spiking, the samples were allowed to equilibrate in the dark for 15 min before extraction. For mycotoxin extraction, 20 mL of methanol/ethyl acetate/water (70:20:10, v/v/v) was added to 3.000 ± 0.003 g of homogenized flour. The mixture was vortexed, agitated for 40 min and centrifuged at 4000 × g for 10 min. The supernatant was transferred to a clean extraction tube and defatted by adding 10 mL of n-hexane. The extract was agitated for 15 min and centrifuged at 4000 × g for 15 min. Following centrifugation, the upper n-hexane layer was removed and discarded, while the remaining defatted extract was retained for further purification. The defatted extract was then divided between liquid-liquid extraction/precipitation and solid-phase extraction (SPE).

A 2.5 mL aliquot of the defatted extract was transferred to a 50 mL extraction tube containing 10 mL dichloromethane/formic acid (95:5, v/v), vortexed and centrifuged at 4000 × g for 10 min. The remaining defatted extract was purified using an amino-SPE cartridge. The cartridge was conditioned twice with 6 mL methanol/ethyl acetate/water (70:20:10, v/v/v), and the conditioning eluate was discarded. The defatted extract was then applied to the conditioned cartridge, and the sample eluate was collected. The extraction tube was rinsed twice with 2 mL extraction solvent, and the rinses were included in the collected fraction. The clarified liquid above the precipitate obtained following dichloromethane/formic-acid cleanup was combined with the amino-SPE eluate. The pooled extract was evaporated at 60 °C under a gentle nitrogen stream. The dried residue was reconstituted in 300 µL of injection solvent and vortexed thoroughly. After adding 200 µL of n-hexane, the mixture was vortexed for 2 min and centrifuged at 3300 × g for 5 min. The entire 500 µL mixture was transferred to a 0.22 µm centrifuge filter and centrifuged at 10,000 × g for 10 min. A 100 µL aliquot of the lower filtrate phase was transferred to an LC vial containing an insert and mixed with 100 µL of injection solvent. The vial was capped, vortexed and gently tapped to remove air bubbles from the bottom of the insert. LC–MS/MS acquisition was performed using analyte-specific multiple reaction monitoring (MRM). The precursor and product ions, collision energies, cone voltages, ionization mode and predicted retention times for all 23 mycotoxins are presented in **Supplementary Table S2**. Quantified results were accompanied by absolute expanded measurement uncertainty, U, expressed in µg/kg. In accordance with the laboratory protocol, U was calculated using a coverage factor of k = 2, corresponding to an approximate 95% level of coverage.

### 2.8 ELISA determination of histamine and allergen-related immunoreactivity in insect samples

Seventeen species-processing preparations representing 10 insect taxa were evaluated for histamine and lysozyme- and crustacean-reactive ELISA signals. Each preparation was extracted and analyzed separately, and no insect materials or extracts were pooled. Duplicate ELISA wells were treated as technical measurements and averaged to obtain one result per preparation. Values outside the applicable quantitative range were reported using < or > rather than extrapolated numerical concentrations. Because the immunoassays measure antibody reactivity rather than human IgE binding, results are described as lysozyme- and crustacean-reactive signals rather than evidence of clinical allergenicity.

#### 2.8.1 Insect sample preparation

Approximately 500 g from each batch of insects evaluated in raw and pan-fried states was obtained from icipe. Each batch was divided into two approximately equal portions: one portion was retained raw, whereas the other was pan-fried. The portions were ground separately to homogeneity and stored at 3 °C until analysis. Samples analyzed in dried form were ground until at least 95% of the material passed through a 20-mesh sieve, and approximately 200 g per species was stored at 3 °C until analysis (**Figure 1**). *Gonimbrasia belina* and *Gynanisa maja* were oven-dried, ground until at least 95% of the material passed through a 20-mesh sieve and stored at 3 °C until analysis. Raw and pan-fried preparations were assigned separate species-processing codes. When they originated from paired portions of the same source batch, they were treated as paired processing preparations rather than independent source-batch replicates. Where applicable, a separately collected later batch was used for histamine analysis, while dried preparations originated from separately prepared batches. **Table 1** aligns assay results by taxon and processing state; an aligned row does not necessarily represent the same physical source batch across all three ELISA endpoints. No insect materials or extracts were pooled.

**Table 1:** ELISA results for individually analyzed insect preparations according to taxon and processing state.

Insect taxon	Processing state	Histamine (µg/kg)	Lysozyme-reactive ELISA result (µg/kg)	Crustacean-reactive ELISA result (µg/kg)
*Bunaea alcinoe*	Fresh	8,947	< 25	> 400
*Bunaea alcinoe*	Pan-fried	10,342	< 25	> 400
*Schistocerca gregaria*	Fresh	< 2,500	< 25	> 400
*Schistocerca gregaria*	Pan-fried	8,947	< 25	> 400
*Gonimbrasia zambesina*	Fresh	< 2,500	< 25	> 400
*Gonimbrasia zambesina*	Pan-fried	< 2,500	< 25	> 400
*Ruspolia differens*	Fresh	3,108	< 25	> 400
*Ruspolia differens*	Pan-fried	3,825	< 25	> 400
*Gryllus bimaculatus*	Fresh	4,354	< 25	> 400
*Scapsipedus icipe*	Fresh	3,694	< 25	> 400
*Scapsipedus icipe*	Pan-fried	5,389	< 25	> 400
*Macrotermes subhyalinus*	Fresh	3,705	< 25	> 400
*Macrotermes subhyalinus*	Pan-fried	3,199	< 25	> 400
*Gonimbrasia belina*	Dried	> 40,000	< 25	382
*Gynanisa maja*	Dried	14,288	< 25	> 400
*Chaoborus* spp.	Raw	30,232	< 25	> 400
*Chaoborus* spp.	Pan-fried	27,310	< 25	> 400

Raw and pan-fried preparations from the same source batch were paired processing samples. Duplicate ELISA wells were technical measurements and were averaged to obtain one result per preparation. Table rows align results by taxon and processing state and do not necessarily represent the same physical source batch across all three ELISA endpoints.

Histamine concentrations were converted from ppm to µg/kg using 1 ppm = 1,000 µg/kg. Results below 2.5 ppm and above 40 ppm were reported as < 2,500 and > 40,000 µg/kg, respectively. Lysozyme- and crustacean-reactive results generated in ppb were expressed as µg/kg using 1 ppb = 1 µg/kg. Results outside their quantitative ranges were reported using inequality signs.

#### 2.8.2 Histamine determination

Histamine was determined using the Veratox^®^ for Histamine competitive direct ELISA (Product No. 9505; AOAC Research Institute Performance Tested Method No. 070703) according to the manufacturer’s instructions (Neogen Corporation, 2018). For wet samples, 10 g of homogenate was added to 90 mL of distilled or deionized water; for dried samples, 10 g was added to 100 mL of water. Each extraction bottle was shaken vigorously for 15–20 s initially and again after each of two successive 5-min intervals. The material was allowed to settle for approximately 30 s and was filtered when necessary. Subsequently, 100 µL of the extract was added to 10 mL of sample-extract diluent and mixed gently.

Before analysis, all reagents were equilibrated to 18–30 °C. Conjugate (100 µL) and 100 µL of each control or diluted sample extract were mixed in red-marked mixing wells. A 100-µL aliquot was transferred to the corresponding antibody-coated well and incubated for 10 min at room temperature, with gentle plate movement during the first 10–20 s. The antibody-coated wells were washed four times, after which 100 µL of substrate was added. Following incubation for 10 min, with gentle plate movement during the first 10–20 s, the reaction was stopped by adding 100 µL of stop solution. Absorbance was measured at 630 nm within 20 min. The assay quantitative range was 2.5 to 40 ppm. Results below or above this range were reported as < 2.5 or > 40 ppm, respectively. No additional dilution and reanalysis was performed for the result above 40 ppm.

#### 2.8.3 Lysozyme- and crustacean-reactive ELISAs

Lysozyme- and crustacean-reactive immunoreactivity was assessed using the AgraQuant^®^ Lysozyme ELISA Test Kit (Item No. 10002090; calibration range, 25–250 µg/kg; manufacturer-stated limit of detection, 2 µg/kg) and AgraQuant^®^ Crustacea ELISA Test Kit (Item No. 10002076; calibration range, 20–400 µg/kg; manufacturer-stated limit of detection, 0.9 µg/kg), respectively (Romer Labs Division Holding GmbH, 2024a, b). For each assay, 1 g of homogenized sample was extracted with 20 mL of the kit-specific extraction buffer according to the manufacturer’s instructions. Standards and sample extracts were added to antibody-coated wells and incubated for 20 min. After washing, conjugate was added and the wells were incubated for 20 min. The wells were washed again, substrate was added, and the plates were incubated for 20 min in the dark. Stop solution was then added, and absorbance was measured at 450 nm using 630 nm as the reference wavelength. For both sandwich ELISAs, analyte concentration was determined from the mean absorbance using the kit standard curve and point-to-point interpolation. The manufacturer’s calibration incorporated the 1:20 solid-sample extraction ratio. Results outside the calibration ranges were not extrapolated; lysozyme-reactive signals below 25 µg/kg were reported as < 25 µg/kg and crustacean-reactive signals above 400 µg/kg were reported as > 400 µg/kg.

### 2.9 Statistical analysis

Fungal-isolate counts were aggregated separately for each insect product, food ingredient, laboratory-prepared formulation and commercial complementary flour and organized into contingency tables according to fungal category. The contingency analyses were based on the 798 recovered fungal isolates rather than the 1,080 culture observations. They therefore evaluated the distribution of recovered isolates among fungal categories and sampled materials, rather than plate-level fungal recovery. Pearson’s chi-square tests of independence were performed using IBM SPSS Statistics, version 25, to evaluate associations between sampled products or product groups and the distribution of recovered fungal isolates among *Aspergillus*, *Penicillium*, *Fusarium* and other genera. For the comparison between insect-based formulations and commercial complementary flours, cellwise Fisher’s exact tests were performed using XLSTAT, version 2023.1.4. The three replicate stock suspensions and four dilution levels were nested within the originating independent batch and were not entered as independent biological samples. The analyses were interpreted as exploratory associations among the isolate-count distributions observed in the sampled products and batches. Because each culture medium was assigned to a different batch and culture occasion, medium and batch effects could not be separated statistically; medium-specific recovery patterns were therefore interpreted descriptively.

Histamine concentrations and lysozyme- and crustacean-reactive ELISA results were analyzed descriptively. The ELISA component was designed as an exploratory screening assessment and did not include multiple independently collected batches within every species-by-processing-and-assay category. Duplicate optical-density readings from the same sample extract were technical measurements and were averaged to obtain one assay-specific result; they were not considered independent biological replicates. Results within the applicable quantitative range were reported in µg/kg, whereas results below or above that range were reported using < or >, respectively. No factorial ANOVA or other inferential ELISA comparison was performed because the design was not a replicated factorial experiment and most lysozyme- and crustacean-reactive results were outside the assays’ quantitative ranges.

The five formulations were predefined through earlier nutritional-formulation and sensory-evaluation studies. The LC-MS/MS storage experiment was an exploratory safety assessment of this fixed product set rather than a confirmatory comparison based on a prespecified effect size. Consequently, no *a priori* statistical power calculation was performed. The three independently prepared bottles per formulation and sampling period provided preparation-level replication, and interpretation was restricted to the evaluated formulations and batches.

## 3 Results

### 3.1 Fungal contaminants of edible insects, contemporary food ingredients, porridge flour ingredients and formulations

Fungal isolation comprised 1,080 inoculated culture observations across 30 material types, from which 798 fungal isolates were recovered. Recovery varied among culture observations: some yielded no recoverable isolate, whereas others yielded one or more morphologically distinct isolates. Morphological assessment, supported by molecular identification of representative purified morphotypes, resulted in assignment to 11 genera*: Aspergillus, Fusarium, Gibberella, Hypocrea, Meyerozyma, Penicillium, Pleurotus, Talaromyces, Trichoderma, Ustilaginoidea* and *Vanrija*. Across all material categories, *Aspergillus* accounted for 55.3% of the 798 recovered culture isolates, followed by *Penicillium* at 26.1%, *Fusarium* at 14.5% and other genera at 4.1% (**Table 2**). Among isolates recovered specifically from insect products, *Aspergillus* accounted for 70.7% (**Table 3**).

**Table 2:** Distribution of fungal isolates recovered from 30 material types (1,080 culture observations; 798 fungal isolates).

^a^ Material category	Aspergillus	Penicillium	Fusarium	Others	Total
Insects (396 culture observations)	239 (70.7)	63 (18.6)	25 (7.4)	11 (3.3)	338 (100)
Food ingredients (252 culture observations)	106 (45.9)	61 (26.4)	57 (24.7)	7 (3.0)	231 (100)
Developed formulations (252 culture observations)	68 (49.3)	48 (34.8)	12 (8.7)	10 (7.2)	138 (100)
Commercial complementary flours (180 culture observations)	28 (30.8)	36 (39.6)	22 (24.2)	5 (5.5)	91 (100)
All materials (1,080 culture observations)	441 (55.3)	208 (26.1)	116 (14.5)	33 (4.1)	798 (100)**

Culture observations comprised all inoculated batch-replicate-dilution combinations, including plates with no recovered isolate. Because a culture observation could yield no isolate, one isolate or more than one morphologically distinct isolate, percentages were calculated from the 798 recovered isolates rather than the 1,080 culture observations. Values are presented as n (%), where n denotes the fungal-isolate count in each table cell.

Each independent material batch was prepared in triplicate. For each replicate stock suspension, the undiluted stock and three serial dilutions (10^-^¹, 10^-^² and 10^-^³) were plated, giving 12 culture observations per batch (3 replicate stock suspensions × 4 dilution levels) and 36 per material type (3 batches × 12 observations). The replicate stock suspensions and dilution levels were nested within the originating batch and were not additional independent batches. Table cells report fungal-isolate counts; values in parentheses are percentages of the corresponding isolate total.

^a^ Independent material batch n = 3 per material type. The three batches were processed on different days, with one batch assigned to half-strength potato dextrose agar, one to full-strength potato dextrose agar and one to Sabouraud dextrose agar. Within each batch, three replicate stock suspensions were prepared and plated at four dilution levels.

Cell entries are fungal-isolate counts; values in parentheses are percentages of the corresponding row total.

Chi-square test of independence based on aggregate fungal-isolate counts:

**Association between material category and fungal category (χ² = 88.784, df = 9, *p* < 0.001).Bold values indicate column totals across the rows presented in the table.

**Table 3:** Fungal isolates recovered from 11 insect products (396 culture observations; 338 fungal isolates).

^a^ Insect	Common name	Aspergillus	Penicillium	Fusarium	Others	Total
Anaphe panda caterpillar	African wild silk moth	14 (77.8)	1 (5.6)	2 (11.1)	1 (5.6)	18 (100)
Bombyx mori	Silkworm pupae	30 (78.9)	8 (21.1)	0 (0)	0 (0)	38 (100)
Gonimbrasia belina-pupae	Mopane pupae	22 (62.9)	8 (22.9)	2 (5.7)	3 (8.6)	35 (100)
Gonimbrasia zambesina-pupae	Mopane pupae	15 (62.5)	8 (33.3)	1 (4.2)	0 (0)	24 (100)
Gynanisa maja	Mopane worms	24 (75)	7 (21.9)	0 (0)	1 (3.1)	32 (100)
Gryllus bimaculatus	Crickets	18 (60)	11 (36.7)	1 (3.3)	0 (0)	30 (100)
Scapsipedus icipe	icipe Crickets	6 (60)	4 (40)	0 (0)	0 (0)	10 (100)
Schistocerca gregaria	Desert locusts	31 (72.1)	2 (4.7)	7 (16.3)	3 (7)	43 (100)
Ruspolia differens	Wild grasshoppers	34 (73.9)	3 (6.5)	8 (17.4)	1 (2.2)	46 (100)
Macrotermes subhyalinus	Winged termites	24 (68.6)	8 (22.9)	2 (5.7)	1 (2.9)	35 (100)
Chaoborus sp.	Lake-flies	21 (77.8)	3 (11.1)	2 (7.4)	1 (3.7)	27 (100)
	Total =396	239 (70.7)	63 (18.6)	25 (7.4)	11 (3.3)	338 (100)*

Each independent insect-product batch was prepared in triplicate. For each replicate stock suspension, the undiluted stock and three serial dilutions (10^-^¹, 10^-^² and 10^-^³) were plated, giving 12 culture observations per batch (3 replicate stock suspensions × 4 dilution levels) and 36 per insect product (3 batches × 12 observations). The replicate stock suspensions and dilution levels were nested within the originating batch and were not additional independent batches. Culture-observation totals include plates with no recovered isolate. Table cells report fungal-isolate counts; values in parentheses are percentages of the corresponding isolate total.

^a^ Independent insect-product batch n = 3 per insect product. The three batches were processed on different days, with one batch assigned to half-strength potato dextrose agar, one to full-strength potato dextrose agar and one to Sabouraud dextrose agar. Within each batch, three replicate stock suspensions were prepared and plated at four dilution levels.

Chi-square test of independence based on aggregate fungal-isolate counts:

*Association between insect product and fungal category (χ² = 51.100, df = 30, p = 0.010).Bold values indicate column totals across the rows presented in the table.

#### 3.1.1 Fungal contaminants of edible insects

Among the insect products, the distribution of recovered isolates among fungal categories differed significantly (χ² = 51.100, *df* = 30, *p* = 0.010; **Table 3**). *Aspergillus* accounted for more than two-thirds of the recovered isolates, followed by *Penicillium* and *Fusarium*. *Fusarium* was not recovered from silkworm pupae, mopane worms or *Scapsipedus icipe*. Other recovered genera were *Hypocrea*, *Meyerozyma*, *Pleurotus*, *Talaromyces* and *Trichoderma*.

#### 3.1.2 Contemporary food ingredients from Western Kenya and their fungal contaminants

The distribution of recovered isolates among fungal categories differed significantly across ingredient types (χ² = 125.987, *df* = 18, *p* < 0.001; **Table 4**). *Aspergillus* was the most frequently recovered genus overall and predominated in grain-amaranth and sweet-potato flours. *Penicillium* predominated in dried mango and milk powder, whereas *Fusarium* predominated in finger millet. Other recovered genera were *Meyerozyma*, *Pleurotus* and *Talaromyces*.

**Table 4:** Fungal isolates recovered from seven food ingredients (252 culture observations; 231 fungal isolates).

^a^ Ingredients	Common name	Aspergillus	Penicillium	Fusarium	Others	Total
Amaranthus cruentus	Grain amaranth seeds	18 (58.1)	8 (25.8)	5 (16.1)	0 (0)	31 (100)
Amaranthus hypochondriacus	Grain amaranth seeds	22 (55)	12 (30)	6 (15)	0 (0)	40 (100)
Mangifera indica	Apple mango	3 (18.8)	12 (75)	1 (6.3)	0 (0)	16 (100)
Whole cow milk powder	Milk powder	2 (15.4)	6 (46.2)	2 (15.4)	3 (23.1)	13 (100)
Eleusine coracana	Finger millet	7 (12.5)	11 (19.6)	35 (62.5)	3 (5.4)	56 (100)
Ipomoea batatas	Orange-fleshed-sweet potato- Kenspot variety	31 (88.6)	1 (2.9)	3 (8.6)	0 (0)	35 (100)
Ipomoea batatas	Orange-fleshed-sweet potato- Kabode variety	23 (57.5)	11 (27.5)	5 (12.5)	1 (2.5)	40 (100)
	Total =252	106 (45.9)	61 (26.4)	57 (24.7)	7 (3.0)	231 (100)**

Each independent ingredient batch was prepared in triplicate. For each replicate stock suspension, the undiluted stock and three serial dilutions (10^-^¹, 10^-^² and 10^-^³) were plated, giving 12 culture observations per batch (3 replicate stock suspensions × 4 dilution levels) and 36 per ingredient type (3 batches × 12 observations). The replicate stock suspensions and dilution levels were nested within the originating batch and were not additional independent batches. Culture-observation totals include plates with no recovered isolate. Table cells report fungal-isolate counts; values in parentheses are percentages of the corresponding isolate total.

^a^ Independent ingredient batch n = 3 per ingredient type. The three batches were processed on different days, with one batch assigned to half-strength potato dextrose agar, one to full-strength potato dextrose agar and one to Sabouraud dextrose agar. Within each batch, three replicate stock suspensions were prepared and plated at four dilution levels.

Chi-square test of independence based on aggregate fungal-isolate counts:

**Association between ingredient type and fungal category (χ² = 125.987, df = 18, *p* < 0.001).Bold values indicate column totals across the rows presented in the table.

#### 3.1.3 Porridge formulations and their fungal contaminants

The porridge formulations and commercial complementary flours are presented in **Table 5**. Across the formulation and commercial-flour groups, *Aspergillus*, *Penicillium* and *Fusarium* were recovered, although their relative distributions varied among individual products. The aggregate fungal-category distribution differed between the developed formulations and commercial complementary flours (χ² = 13.929, *df* = 3, *p* = 0.003). Cellwise Fisher exact tests showed that *Aspergillus* was more frequently recovered from the developed formulations (*p* = 0.006), whereas *Fusarium* was more frequently recovered from the commercial complementary flours (*p* = 0.002). *Penicillium* occurred in both groups. The other genera recovered from the developed formulations were *Talaromyces*, *Trichoderma* and *Vanrija*, while *Gibberella*, *Pleurotus* and *Talaromyces* were recovered from the commercial complementary flours.

**Table 5:** Fungal isolates recovered from seven developed formulations and five commercial complementary flours (432 culture observations; 229 fungal isolates).

^a^ Enriched insects	Common name	Aspergillus	Penicillium	Fusarium	Others	Total
With whole cow milk powder						
S. icipe	icipe crickets (CR+M)	15 (65.2)	6 (26.1)	0 (0)	2 (8.7)	23 (100)
Without insect	Milk powder (M+O)	8 (61.5)	5 (38.5)	0 (0)	0 (0)	13 (100)
Without whole cow milk powder						
S. gregaria	Desert locust (LOC+O)	1 (6.7)	12 (80)	1 (6.7)	1 (6.7)	15 (100)
R. differens	Wild grasshopper (GH+O)	32 (69.6)	3 (6.5)	6 (13)	5 (10.9)	46 (100)
Chaoborus sp.	Lake-flies (LKF+O)	5 (25)	10 (50)	4 (20)	1 (5)	20 (100)
S. icipe	icipe crickets (CR+O)	3 (25)	7 (58.3)	1 (8.3)	1 (8.3)	12 (100)
M. subhyalinus	Winged termites (TMW-O)	4 (44.4)	5 (55.6)	0 (0)	0 (0)	9 (100)
Total =252		68 (49.3**)	48 (34.8)	12 (8.7**)	10 (7.2)	138 (100)
^a^ Commercial flours	Composition of the flours					
Flour – A	Finger millet,/wheat/soya flours/sour/sugar.	1 (3.7)	21 (77.8)	3 (11.1)	2 (7.4)	27 (100)
Flour - B	Whole maize/soya beans/sorghum flours, sugar.	13 (76.5)	4 (23.5)	0 (0)	0 (0)	17 (100)
Flour - C	Finger millet/grain amaranth seeds/groundnuts/soya beans/refined maize (fermented) flours.	1 (6.3)	0 (0)	14 (87.5)	1 (6.3)	16 (100)
Flour - D	Finger millet/sorghum/cassava/refined maize flour, souring agents.	11 (57.9)	7 (36.8)	1 (5.3)	0 (0)	19 (100)
Flour - E	Finger millet/sorghum/cassava/refined maize flour, souring agents.	2 (16.7)	4 (33.3)	4 (33.3)	2 (16.7)	12 (100)
Total =180		28 (30.8)**	36 (39.6)	22 (24.2)**	5 (5.5)	91 (100)
Total fungal contaminants from enriched insect flours + commercial flours (Total = 432)		96 (41.9)	84 (36.7)	34 (14.8)	15 (6.6)	229 (100)*

The developed formulations contributed 252 culture observations and 138 isolates, whereas the commercial flours contributed 180 culture observations and 91 isolates.

Base ingredients in LOC+O, GH+O, LKF+O, CR+O, TMW-O, CR+M, M+O; Amaranthus hypochondriacus (Grain amaranth seeds), Ipomoea batatas (Orange-fleshed-sweet potato- Kabode variety), Mangifera indica (Apple mango).

Each independent formulation or commercial-flour batch was prepared in triplicate. For each replicate stock suspension, the undiluted stock and three serial dilutions (10^-^¹, 10^-^² and 10^-^³) were plated, giving 12 culture observations per batch (3 replicate stock suspensions × 4 dilution levels) and 36 per product (3 batches × 12 observations). The replicate stock suspensions and dilution levels were nested within the originating batch and were not additional independent batches. Culture-observation totals include plates with no recovered isolate. Table cells report fungal-isolate counts; values in parentheses are percentages of the corresponding isolate total.

^a^ Independent formulation or commercial-flour batch n = 3 per product. The three batches were processed on different days, with one batch assigned to half-strength potato dextrose agar, one to full-strength potato dextrose agar and one to Sabouraud dextrose agar. Within each batch, three replicate stock suspensions were prepared and plated at four dilution levels.

Chi-square test of independence based on aggregate fungal-isolate counts:

*Association between formulation group and fungal category (χ² = 13.929, df = 3, p = 0.003).

**Cellwise Fisher’s exact tests indicated significant differences for Aspergillus (p = 0.006) and Fusarium (p = 0.002) between insect-based formulations and commercial complementary flours.

#### 3.1.4 Fungal contaminants of food ingredients, enriched insect formulations and commercial complementary flour

Across the four material groups, the distribution of recovered isolates among *Aspergillus*, *Penicillium*, *Fusarium* and other genera differed significantly (χ² = 88.784, *df* = 9, *p* < 0.001; **Table 2**). *Aspergillus* was the predominant fungal category among insects, food ingredients considered collectively and enriched insect formulations, whereas *Penicillium* was predominant in commercial complementary flours.

#### 3.1.5 Exploratory PCR screening for mycotoxin-associated markers

Among the 44 isolates provisionally classified as *Aspergillus flavus*-like morphotypes, 32 produced the expected *aflD* amplicon, 23 produced the expected *aflQ* amplicon and 19 were positive for both markers. Among the 22 *Fusarium*-like isolates, 10 produced the expected *VERTF* amplicon and 19 produced the expected *FUM1* amplicon. These results demonstrate detection of the targeted molecular markers in the selected cultures but do not establish gene expression or mycotoxin production (**Supplementary Table S6**).

### 3.2 Mycotoxins occurrence in insect-fortified flours during storage

Mycotoxin results for the five formulations, comprising four insect-fortified formulations and one non-insect control, during the three-month storage period are presented in **Table 6**. Six of the 23 mycotoxins screened were detected: 3-acetyldeoxynivalenol (3-ADON), aflatoxin G1 (AFG1), sterigmatocystin (STERIG), and fumonisins B1 (FB1), B2 (FB2), and B3 (FB3). Only AFG1 and FB1 yielded results above their applicable limits of quantification (LOQs). AFG1 was quantified in one M+O replicate at month 1 at 4.20 ± 1.91 µg/kg. Quantifiable FB1 concentrations ranged from 20.31 to 86.88 µg/kg; all remaining confirmed FB1 detections were below the LOQ. The single 3-ADON and FB3 detections and all STERIG and FB2 detections were below their respective LOQs and are therefore reported as < LOQ rather than as numerical concentrations. The corresponding expanded measurement uncertainties for quantified results are presented in **Supplementary Table S1**.

**Table 6:** Detection and quantification status of mycotoxins in insect-fortified and control complementary flour formulations during three months of storage.

Formulation	Common name	Storage period	3-ADON	AFG1	STERIG	FB1	FB2	FB3
With whole-cow-milk powder								
dewinged termites (*M. subhyalinus*) (TM+M)	Dewinged termite	Fresh (month 0)	–	–	–	D < LOQ	D < LOQ	–
		Month 1	–	–	D < LOQ	D < LOQ	D < LOQ	–
		Month 2	–	–	–	D < LOQ	D < LOQ	–
		Month 3	–	–	–	Q	–	–
*R. differens* (GH+M)	Wild grasshopper	Fresh (month 0)	–	–	–	D < LOQ	–	–
		Month 1	–	–	–	D < LOQ	–	–
		Month 2	–	–	–	D < LOQ	–	–
		Month 3	–	–	–	Q	–	–
Without whole-cow-milk powder								
*M. subhyalinus* (TM+O)	Dewinged termite	Fresh (month 0)	–	–	–	D < LOQ	D < LOQ	–
		Month 1	D < LOQ	–	–	D < LOQ	D < LOQ	–
		Month 2	–	–	D < LOQ	Q	–	–
		Month 3	–	–	–	Q	–	–
*R. differens* (GH+O)	Wild grasshopper	Fresh (month 0)	–	–	–	D < LOQ	–	–
		Month 1	–	–	–	D < LOQ	–	–
		Month 2	–	–	–	D < LOQ	D < LOQ	–
		Month 3	–	–	–	Q	–	–
Control formulation								
Without insect (M+O)	Non-insect control	Fresh (month 0)	–	–	–	D < LOQ	–	–
		Month 1	–	Q	–	D < LOQ	–	–
		Month 2	–	–	–	D < LOQ	–	–
		Month 3	–	–	–	Q	D < LOQ	D < LOQ

Q, quantified: at least one of the three independently prepared formulation replicates contained the analyte at or above the applicable limit of quantification (LOQ).

D < LOQ, detected below the LOQ: the analyte was confirmed in at least one replicate, but no replicate yielded a concentration at or above the LOQ. –, not detected in any replicate.

Results are summarized by formulation–storage combination; replicate-level results and expanded measurement uncertainties are presented in **Supplementary Tables** 1 and **S2**.

Method LOQs (µg/kg) were: 3-ADON, 50; AFG1, 2.5; STERIG, 2.5; FB1, 20; FB2, 35; and FB3, 40.

TM+M, dewinged termite formulation with milk powder; GH+M, wild-grasshopper formulation with milk powder; TM+O, dewinged termite formulation without milk powder; GH+O, wild-grasshopper formulation without milk powder; M+O, control formulation without insect.

FB1 was detected in all five formulations at each sampling time. Quantifiable concentrations occurred more frequently at the later storage times, and the highest concentration was recorded after three months. However, the temporal pattern was not uniformly monotonic across the formulations. Because grain amaranth (*Amaranthus hypochondriacus*) was common to all formulations, it may have contributed to the widespread FB1 occurrence; nevertheless, the source cannot be assigned to a particular ingredient because the individual ingredient batches were not analyzed separately.

KS EAS 72:2024 establishes maximum levels of 3 µg/kg for aflatoxin B1 and 5 µg/kg for total aflatoxins in processed cereal-based foods for older infants and young children (Kenya Bureau of Standards, 2024). The detected AFG1 concentration contributes to total aflatoxins but is not directly comparable with the AFB1-specific maximum. Commission Regulation (EU) 2023/915 establishes a maximum AFB1 level of 0.10 µg/kg for baby food and processed cereal-based food intended for infants and young children. The Regulation also establishes a maximum of 200 µg/kg for the sum of FB1 and FB2 specifically in maize-containing baby food and processed maize-based food intended for infants and young children. Both European Union maximum levels are expressed on a dry-matter basis. Because the present formulations were not maize-based, the European Union fumonisin maximum is presented only as a contextual benchmark (Kenya Bureau of Standards, 2024; European Commission, 2023).

### 3.3 Histamine concentrations and allergen-related ELISA reactivity in edible insects

Seventeen species-processing preparations representing 10 insect taxa were evaluated across the histamine, lysozyme-reactive and crustacean-reactive ELISA endpoints (**Table 1**). Among results within the quantitative range, histamine concentrations ranged from 3,108 to 30,232 µg/kg. Three results were below the limit of quantification (< 2,500 µg/kg), whereas one result exceeded the upper quantitative limit and was reported as > 40,000 µg/kg.

Lysozyme-reactive signals were below the lower quantification limit (< 25 µg/kg) in all 17 insect preparations. Crustacean-reactive signals exceeded the upper quantification limit (> 400 µg/kg) in 16 preparations, whereas the oven-dried *Gonimbrasia belina* preparation yielded a quantifiable result of 382 µg/kg. These inequalities represent analytical quantification boundaries and should not be interpreted as dietary-intake or health-based safety thresholds.

## 4 Discussion

This study provides the first detailed assessment of fungal contamination in edible insects commonly consumed in Africa, related ingredients, insect-fortified and non-insect fortified porridges, and commercial complementary formulations. Fungi occurred in 94% of insect and ingredient samples and over 70% of composite flours, indicating widespread contamination. *Aspergillus* was the most common genus, followed by *Penicillium* and *Fusarium*, consistent with earlier regional reports in insects and cereals from Kenya, Uganda and Tanzania (Muthomi et al., 2008; Okoth, 2016; Rose et al., 2017). Similar contamination patterns have been documented in a wide range of African staples, including grain amaranth, mango, milk, wheat, maize, soybean, cassava, sorghum, peanuts and sugar (Pitt and Hocking, 2009). In this study, fungal isolates were recovered from formulations both with and without milk powder; however, the exploratory design did not permit attribution of fungal occurrence to individual ingredients, and therefore no conclusion could be drawn about whether milk powder was a major source of *Aspergillus*.

The high prevalence of *Aspergillus* and *Fusarium* is a major food safety concern because of aflatoxins and fumonisins, which are carcinogenic, immunotoxic and associated with other adverse health effects (Mafe and Büsselberg, 2024; Navale et al., 2021). Warm, humid climates; delayed drying; poor storage; and limited preservation and heat-treatment technologies in low-resource settings promote fungal growth and mycotoxin accumulation (Mafe and Büsselberg, 2024). Insect surfaces and gut contents can act as reservoirs for toxigenic fungi when hygiene and processing are inadequate, especially when field-harvested or sun-dried under open conditions that increase exposure to airborne spores (Schrögel and Wätjen, 2019). Although insects themselves generally do not accumulate high internal mycotoxin levels, surface and gut contamination can persist, particularly if the gut is not purged before processing, and the rearing substrate and storage environment remain critical determinants of contamination (Schrögel and Wätjen, 2019). Mycotoxins are heat stable and often survive conventional processing, including in packaged foods; co-contamination with multiple toxins, as well as masked or modified forms undetectable by standard methods, further elevates health risks and complicates risk assessment (Navale et al., 2021; Pereira et al., 2022).

*Penicillium* and *Fusarium* were also frequently isolated from insects, ingredients, insect-enriched porridges and commercial complementary flours. The recovery of *Fusarium* from finger millet and some commercial complementary flours is consistent with its established association with cereals such as millet, maize and sorghum (Muthomi et al., 2008; Rose et al., 2017), as well as in millet, grain amaranth, mango, milk, sweet potato, soybean, peanuts and cassava (Pitt and Hocking, 2009). *Penicillium* species, often overlooked in insect matrices, are likewise common contaminants of grain amaranth, mango, milk, wheat, maize, peanuts, cassava and sorghum under diverse storage and market conditions (Pitt and Hocking, 2009). Some *Penicillium* and *Fusarium* species produce patulin, citrinin, fumonisins and trichothecenes, adding to the public health burden (Adekoya et al., 2017).

Multiple mycotoxins frequently co-occur in staple African foods, including insect-fortified and cereal-based porridges, even after processing and packaging, raising concerns about cumulative exposure in infants and young children, who have developing systems and higher intake per body weight (Chilaka and Mally, 2020; Ojuri et al., 2019; Pereira et al., 2022). Evidence from northern Tanzania further demonstrates the vulnerability of this population: dietary exposure to aflatoxins and fumonisins increased as maize-based complementary foods were introduced to infants younger than six months (Magoha et al., 2016). Studies from Nigeria and other African countries show that 42–93% of complementary foods including household and industrial, test positive for mycotoxins, and about 47% of infants and young children are co-exposed to two to four toxins, including aflatoxins, fumonisins, citrinin and ochratoxin A. Many samples, particularly household preparations, exceed European Union and international safety thresholds, with aflatoxin and fumonisin exposures often above recommended limits (Chilaka and Mally, 2020). Limited caregiver awareness and weak enforcement of food safety standards exacerbate exposure, underscoring the need for targeted education and stronger regulatory implementation to protect vulnerable groups (Mollay et al., 2021; Ojuri et al., 2019).

Mycotoxin contamination also has substantial socioeconomic consequences through crop and product losses, food rejection, testing and control costs, and restrictions on market access and international trade (Mafe and Büsselberg, 2024). Climate change may modify mycotoxin risk by altering temperature, precipitation, drought and other environmental conditions that influence fungal ecology and mycotoxin production, although the magnitude and direction of these effects vary among fungal species, toxins, crops and regions (Kos et al., 2023). Effective control therefore requires preventive measures throughout ingredient sourcing, processing and storage, supported by routine monitoring, surveillance, training and appropriate technologies. The frequent isolation of *Aspergillus*, *Penicillium* and *Fusarium* in the present study reinforces the need to integrate fungal and mycotoxin control into emerging edible-insect and insect-fortified complementary-food value chains in Africa.

Across the five formulations, six mycotoxins were confirmed: 3-ADON, AFG1, STERIG, FB1, FB2 and FB3. Only AFG1 and FB1 were quantified above their applicable operational LOQs; the remaining confirmed occurrences were below the respective LOQs and were treated as trace-level detections rather than reported as numerical concentrations. FB1 occurred in all five formulations, including the non-insect control, while the AFG1 and FB3 detections occurred in the control formulation. The findings therefore do not support attribution of the detected mycotoxins specifically to the insect ingredients. Grain amaranth or other ingredients common to the formulations may have contributed, but the source cannot be assigned because the individual ingredient batches were not analyzed separately for mycotoxins.

Under Commission Regulation (EU) 2023/915, the maximum level for the sum of FB1 and FB2 in baby food containing maize and processed maize-based food intended for infants and young children is 200 µg/kg on a dry-matter basis. Because the complementary flour formulations analyzed for mycotoxins were grain-amaranth-based and contained no maize, this maize-specific value is presented only as a contextual comparator and not as a directly applicable compliance criterion. Nevertheless, the occurrence of both quantifiable results and confirmed detections below the LOQ supports continued monitoring of fungal metabolites in complementary-food formulations intended for infants and young children (European Commission, 2023).

The detection of *Fusarium*-associated metabolites, including 3-ADON and fumonisins, and *Aspergillus*-associated metabolites, including AFG1 and sterigmatocystin, was qualitatively compatible with the recovery of these genera across the study materials (Pandey et al., 2023). However, individual fungal isolates were not tested for mycotoxin production, and the culture and LC–MS/MS results were not linked at isolate level. The recovered genera therefore cannot be identified as the direct sources of the detected metabolites, particularly because mycotoxin-production capacity varies among fungal species and strains and is influenced by environmental conditions. Previous studies nevertheless demonstrate that edible insects can acquire fungal and mycotoxin contamination during handling and storage. Mpuchane et al. (1996) documented fungi associated with *Imbrasia belina* in Botswana. Musundire et al. (2016) detected AFB1 at 0.50 and 0.59 µg/kg in traditionally handled unprocessed and processed edible stink bugs, respectively, whereas AFB1 was not detected in insects collected and stored in clean zip-lock bags. In Zambia, Kachapulula et al. (2018) reported mean aflatoxin concentrations of 11, 12 and 24 µg/kg in *Gynanisa maja*, *Gonimbrasia zambesina* and *Macrotermes falciger*, respectively. These studies support careful control of substrates, handling and storage and routine mycotoxin surveillance in edible-insect foods.

The ingredients used in the present formulations were dried before milling, and the flours were stored in tightly sealed plastic bottles at approximately 22 °C. Ambient relative humidity was not controlled or continuously monitored and was estimated at approximately 45%. These conditions are consistent with a low-moisture flour system, whose stability depends on product moisture content, water activity and packaging-barrier properties (Gichau et al., 2020; Maiyo et al., 2025). The temporal differences were therefore interpreted as storage-associated patterns and not as direct evidence of humidity-driven fungal growth or *de novo* mycotoxin production (Kiaitsi et al., 2025).

Detection of the *aflD* and *aflQ* aflatoxin-biosynthesis markers, the *FUM1* fumonisin-biosynthesis marker and the *VERTF* intergenic-spacer profile in selected fungal cultures provides biological context for the recovery of *Aspergillus* and *Fusarium* and for the independent LC–MS/MS detection of AFG1 and fumonisins in the formulations (Patiño et al., 2004; Bluhm et al., 2004; Okoth et al., 2012). However, endpoint PCR established only the presence of the targeted DNA markers, whereas LC–MS/MS was performed on formulation extracts rather than on the individual fungal cultures. The two analyses were not linked isolate by isolate; consequently, a marker-positive culture cannot be identified as the direct source of a mycotoxin detected in a formulation.

Across the insect sample preparations, histamine results included both quantifiable and censored measurements. All lysozyme-reactive ELISA signals were < 25 µg/kg, while 16 crustacean-reactive signals were > 400 µg/kg and the remaining result was 382 µg/kg. Among results within the histamine assay’s quantitative range, concentrations ranged from 3,108 to 30,232 µg/kg (3.108–30.232 mg/kg). Three results were below the limit of quantification (< 2,500 µg/kg). The oven-dried *Gonimbrasia belina* result exceeded the upper quantitative limit and was therefore reported as > 40,000 µg/kg; its exact concentration could not be established. The highest histamine concentrations within the quantitative range were recorded in raw and pan-fried lake flies (*Chaoborus* spp.), at 30,232 and 27,310 µg/kg, respectively. Histamine intoxication is a toxicological foodborne reaction that can produce allergy-like symptoms but does not constitute evidence of IgE-mediated food allergy.

Histidine is the biochemical precursor of histamine, and microorganisms possessing histidine decarboxylase can convert free histidine into histamine under inadequate time–temperature control. European Union criteria therefore regulate histamine concentrations in fishery products derived from fish species associated with high histidine concentrations; histidine itself is not the regulated analyte. Under Commission Regulation (EC) No 2073/2005, the nine-unit sampling plan for these products specifies m = 100 mg/kg and M = 200 mg/kg. For enzyme-matured fishery products in brine, the corresponding values are m = 200 mg/kg and M = 400 mg/kg, whereas fermented fish sauce has a criterion of 400 mg/kg (European Commission, 2005, 2013). International histamine limits for fish products range from approximately 50 to 400 mg/kg and employ differing sampling plans (DeBeer et al., 2021). These criteria are specific to fishery products and cannot be applied directly to edible insects or insect-based complementary foods. Accordingly, the present results were interpreted descriptively rather than as evidence of regulatory compliance or product safety.

Chomchai and Chomchai (2018) reported histamine concentrations of 97.3 mg/kg in fried grasshoppers and 76.6 mg/kg in fried silkworm pupae during an outbreak investigation in Thailand. In an earlier Thai outbreak, one silkworm-pupa specimen contained 875 mg/kg histamine (Mungaomklang et al., 2009). These outbreak-associated concentrations exceeded the values quantified within the analytical range in the present study. However, direct comparison with the *G. belina* sample was not possible because its result was reported only as > 40 mg/kg, and comparisons between studies should be interpreted cautiously because the analytical methods and sample matrices differed. Chumjai et al. (2023) subsequently reported that storage at 4 °C or −20 °C delayed histamine accumulation in raw and cooked crickets compared with room-temperature storage. They also found that more than 95% of crickets obtained from 32 farms in Thailand contained < 50 mg/kg histamine and that boiling duration affected histamine concentrations. These findings support prompt post-harvest temperature control and the development of insect-specific sampling, analytical and risk-management guidance.

All 17 lysozyme-reactive ELISA determinations were below the assay’s lower quantification limit (< 25 µg/kg). Lysozymes and lysozyme-like proteins nevertheless have well-established biological functions in insects. Lysozyme-like activity has been investigated as a component of immune defense in *Gryllus texensis* (Adamo, 2004), while a c-type lysozyme expressed in the fat body, hemocytes and other tissues of *Schistocerca gregaria* showed inhibitory activity against Gram-positive and Gram-negative bacteria (Mohamed et al., 2016). Active lysozymes have also been isolated from the hemolymph of lepidopteran larvae, including *Bombyx mori* (Yu et al., 2002), whereas immune-inducible antibacterial proteins resembling lysozymes but lacking classical muramidase activity have been described in *B. mori* and *Antheraea mylitta* (Gandhe et al., 2007). In the termite *Reticulitermes speratus*, lysozyme additionally functions as a component of the egg-recognition signal (Matsuura et al., 2007).

Hen’s-egg lysozyme is a recognized food allergen, and residual egg-derived lysozyme in foods may retain immunological reactivity and trigger allergic reactions in susceptible egg-allergic individuals (Weber et al., 2009; Lambré et al., 2023). However, evidence concerning hen’s-egg lysozyme does not itself establish IgE cross-reactivity with insect lysozymes or lysozyme-like proteins. The present results should therefore be interpreted as lysozyme-reactive ELISA signals rather than confirmation of a particular insect protein or evidence of clinical allergenicity. Protein characterization and IgE-binding or inhibition studies would be required to determine the relevance of these signals to consumers.

Crustacean-reactive ELISA signals exceeded the assay’s upper quantification limit (> 400 µg/kg) in 16 of the 17 insect preparations; the oven-dried *Gonimbrasia belina* preparation yielded 382 µg/kg. These results represent crustacean-reactive ELISA signals rather than exact concentrations of a defined allergenic protein, and 400 µg/kg is an analytical boundary rather than a health-based safety threshold. More broadly, edible-insect proteins may induce primary sensitization or cross-react with homologous allergens from crustaceans and house-dust mites. Tropomyosin and arginine kinase are among the arthropod proteins most frequently implicated in this cross-reactivity (EFSA NDA Panel, 2021; Yang et al., 2023; Alejandro Ruiz et al., 2025).

Tropomyosin provides a biologically plausible explanation for at least part of the observed analytical cross-reactivity, although the reacting proteins were not identified in the present study. Kamemura et al. (2019) reported a strong correlation between shrimp- and *Gryllus bimaculatus*-specific IgE recognition and identified tropomyosin as a major shared allergen, indicating that some shrimp-sensitized individuals may recognize cricket proteins. Similarly, Leung et al. (1996) demonstrated IgE recognition of grasshopper proteins by sera from shrimp-allergic patients and identified conserved tropomyosin epitopes as an important basis for cross-reactivity among invertebrates. In *Acheta domesticus*, De Marchi et al. (2021) identified tropomyosin as the principal IgE-binding protein, demonstrated cross-reactivity with shrimp tropomyosin and found that cricket tropomyosin remained stable during simulated gastrointestinal digestion; baking also partially protected IgE-binding proteins from proteolysis. Evidence reviewed by de Gier and Verhoeckx (2018) further indicates that processing may decrease, preserve or enhance insect-protein immunoreactivity depending on the species, protein and treatment, and therefore cannot be assumed to eliminate allergenic potential.

Commercial crustacean ELISA results in insect products may also be strongly method-dependent. Karnaneedi et al. (2024) found marked disagreement between two commercial crustacean-allergen kits: one reported apparent crustacean-allergen equivalents in all nine cricket- and black-soldier-fly-based products, including 12.2–610.8 ppm in cricket products and 17,206 ppm in a black-soldier-fly sprinkle, whereas the second kit did not quantify most products. Their proteomic and immunological analyses nevertheless identified tropomyosin and several additional IgE-binding proteins in the insect extracts. These findings reinforce the need to report the present measurements as crustacean-reactive ELISA signals and support clear allergen communication, further protein characterization and IgE-based assessment of edible insects, particularly for consumers with crustacean allergy. Continued monitoring is especially important when insects are intended for incorporation into complementary foods. The ELISA component was exploratory and did not include multiple independent batches within every species-processing category. Confirmation using multiple independently collected batches within each species and processing category is required.

## 5 Conclusions

Edible insects are a promising, sustainable and nutrient-rich food source, but their safety depends on species, rearing, processing and storage. This exploratory study documented culturable fungal contaminants in edible-insect products, food ingredients, prepared formulations and commercial complementary flours, with *Aspergillus, Penicillium* and *Fusarium* predominating. The findings support improved hygienic processing, ingredient control and routine fungal and mycotoxin surveillance. They should not, however, be interpreted as estimates of population-level prevalence or as direct evidence that the recovered fungal isolates produced the detected mycotoxins. Confirmation across independent production batches and settings, together with controlled storage studies and exposure assessment, is required before broader risk or safety conclusions can be drawn.

The histamine and allergen-related findings identify additional food-safety considerations. Histamine was quantifiable in several insect preparations, while lysozyme-reactive ELISA results were below the assay’s lower quantification limit and crustacean-reactive signals exceeded the assay’s upper quantification limit in 16 of 17 preparations. These findings support appropriate post-harvest handling, continued assessment of allergen-related cross-reactivity, clear allergen communication and targeted food-safety guidance for edible insects, particularly when intended for incorporation into complementary foods.

## Data Availability

The data supporting the conclusions of this article are included in the article and its **Supplementary Material**. Molecular-identification metadata supporting the genus-level assignments are provided in **Supplementary Table S3**. The 273 ITS sequences are provided in **Supplementary Data S4** and deposited in the NCBI GenBank Nucleotide database under accession numbers PZ835255–PZ835291 (https://www.ncbi.nlm.nih.gov/nuccore/?term=PZ835255%3APZ835291%5Baccn%5D) and PZ863930–PZ864165 (records begin at https://www.ncbi.nlm.nih.gov/nuccore/PZ863930). The seven LSU D1/D2 sequences are provided in Supplementary Data S5 and deposited under accession numbers PZ835531–PZ835537 (https://www.ncbi.nlm.nih.gov/nuccore/?term=PZ835531%3APZ835537%5Baccn%5D).
