# Supplementary material for: Fungal contamination, mycotoxins, histamine, and allergen-related immunoreactivity in edible insects and insect-fortified complementary foods
Source: Front Fungal Biol. 2026 Sep 14;7:1798430. doi: 10.3389/ffunb.2026.1798430 (PMC13617097; doi:10.3389/ffunb.2026.1798430)
Supplement: Supplementary file 1 [file Table1.docx]

**Supplementary Material**

**Table S1**. Mean of sample-level expanded uncertainties, U (µg/kg)

**A. Quantifiable results**

| Analyte | Formulation | Storage period | Q (*n*/3) | D<LOQ (*n*/3) | NC (*n*/3) | Quantifiable range (µg/kg) | Mean ± SD (µg/kg) | Mean expanded U (µg/kg) |
| --- | --- | --- | --- | --- | --- | --- | --- | --- |
| AFG1 | M+O | Month 1 | 1 | 0 | 2 | 4.20 | 4.20¹ | 1.91 |
| FB1 | M+O | Month 3 | 2 | 1 | 0 | 38.91–52.46 | 45.69 ± 9.58 | 19.10 |
| FB1 | GH+M | Month 3 | 2 | 0 | 1 | 60.66–86.88 | 73.77 ± 18.54 | 30.84 |
| FB1 | GH+O | Month 3 | 2 | 0 | 1 | 21.22–23.15 | 22.19 ± 1.36 | 9.28 |
| FB1 | TM+M | Month 3 | 3 | 0 | 0 | 25.97–35.50 | 31.42 ± 4.91 | 13.14 |
| FB1 | TM+O | Month 2 | 2 | 1 | 0 | 20.31–21.85 | 21.08 ± 1.09 | 8.82 |
| FB1 | TM+O | Month 3 | 3 | 0 | 0 | 30.77–32.47 | 31.53 ± 0.86 | 13.18 |

¹SD was not calculated because only one replicate contained quantifiable AFG1.

**B. Confirmed detections below the LOQ**

| Analyte | Formulation–storage combinations with confirmed detections below the LOQ |
| --- | --- |
| 3-ADON | TM+O, month 1 (1/3) |
| STERIG | TM+M, month 1 (1/3); TM+O, month 2 (3/3) |
| FB1 | M+O: fresh (3/3), month 1 (3/3), month 2 (2/3), month 3 (1/3); GH+M: fresh (2/3), month 1 (2/3), month 2 (2/3); GH+O: fresh (1/3), month 1 (2/3), month 2 (2/3); TM+M: fresh (2/3), month 1 (2/3), month 2 (3/3); TM+O: fresh (2/3), month 1 (3/3), month 2 (1/3) |
| FB2 | M+O, month 3 (2/3); GH+O, month 2 (1/3); TM+M: fresh (1/3), month 1 (1/3), month 2 (1/3); TM+O: fresh (2/3), month 1 (1/3) |
| FB3 | M+O, month 3 (1/3) |

This value is the arithmetic mean of the absolute expanded uncertainties reported for the individual quantified results and is not the expanded uncertainty of the group mean.

Q, quantified and confirmed at or above the applicable LOQ;

D<LOQ, confirmed detection below the LOQ; NC, no confirmed detection. Each formulation–storage combination comprised three independently prepared formulation replicates. Means, ranges and SDs were calculated only from results at or above the LOQ. Below-LOQ estimates were not included in concentration calculations.

Mean expanded U is the arithmetic mean of the sample-level absolute expanded measurement uncertainties and is expressed in µg/kg, not as a percentage. The relative expanded measurement uncertainties used to calculate sample-level U were 36.60% for 3-ADON, 45.45% for AFG1, 43.94% for STERIG, 41.81% for FB1, 9.00% for FB2 and 41.16% for FB3.

Abbreviations: 3-ADON, 3-acetyldeoxynivalenol; AFG1, aflatoxin G1; STERIG, sterigmatocystin; FB1, fumonisin B1; FB2, fumonisin B2; FB3, fumonisin B3; M+O, control formulation without insect; GH+M, *R. differens* formulation with milk powder; GH+O, *R. differens* formulation without milk powder; TM+M, *M. subhylinus* formulation with milk powder; TM+O, *M. subhylinus* formulation without milk powder; LOQ, limit of quantification; U, expanded measurement uncertainty.

**Table S2.** LC–MS/MS multiple-reaction-monitoring parameters specified for the 23-analyte RES-Sorghum reference method and the internal standards DOM and ZAN.

| **Component** | **Ionisation mode** | **Precursor**  **ion (m/z)** | **Fragment**  **ions (m/z)** | **Collision**  **Energy (eV)** | **Cone**  **Voltage (V)** | **Predicted retention time (min)** |
| --- | --- | --- | --- | --- | --- | --- |
| NIV | ESI^+^ | 313 | 124.96*  177.21 | 10  15 | 23 | 3.18 |
| DON | ESI^+^ | 297 | 203.34*  249.47 | 12  13 | 26 | 4.29 |
| FUS-X | ESI^+^ | 355.1 | 175.28*  247.27 | 11  9 | 22 | 5.26 |
| NEO | ESI^+^ | 400.1 | 305.26*  365.07 | 13  10 | 20 | 5.39 |
| DOM | ESI^+^ | 281.2 | 109.21*  137.19 | 18  25 | 23 | 5.48 |
| 3-AcDON | ESI^+^ | 339.2 | 231.23*  203.18 | 10  10 | 23 | 6.47 |
| 15-AcDON | ESI^+^ | 339.1 | 137.1*  321.2 | 10  8 | 24 | 6.49 |
| AFG_2_ | ESI^+^ | 331 | 313.15*  245.3 | 24  30 | 46 | 6.99 |
| AFG_1_ | ESI^+^ | 328.85 | 311.17*  243.36 | 25  20 | 43 | 7.21 |
| AFB_2_ | ESI^+^ | 315 | 287.3*  259.4 | 25  29 | 50 | 7.60 |
| AFB_1_ | ESI^+^ | 313 | 285.27*  241.42 | 21  34 | 47 | 7.87 |
| DAS | ESI^+^ | 384.2 | 307.28*  247.27 | 11  15 | 19 | 7.97 |
| ALT | ESI^+^ | 293.1 | 257.19*  275.16 | 14  10 | 24 | 8.09 |
| ROQC | ESI^+^ | 390.2 | 193.3*  322.24 | 26  21 | 40 | 8.77 |
| HT-2 | ESI^+^ | 441.9 | 215.27*  323 | 10  12 | 16 | 9.26 |
| FB_1_ | ESI^+^ | 722.5 | 334.36*  352.26 | 37  36 | 51 | 9.35 |
| AOH | ESI^+^ | 259.1 | 185.17*  213.15 | 30  25 | 53 | 10.02 |
| T-2 | ESI^+^ | 484.3 | 305.21*  245.21 | 12  12 | 12 | 10.17 |
| FB_3_ | ESI^+^ | 706.4 | 336.3*  354.0 | 35  29 | 51 | 10.53 |
| OTA | ESI^+^ | 404 | 239.13*  101.9 | 22  35 | 24 | 11.14 |
| ZAN | ESI^+^ | 321.2 | 303.2*  189.22 | 14  21 | 27 | 11.19 |
| ZEN | ESI^+^ | 319.2 | 301.24*  238.34 | 12  18 | 22 | 11.50 |
| STERIG | ESI^+^ | 325 | 310.16*  281.33 | 35  25 | 44 | 11.73 |
| FB_2_ | ESI^+^ | 706.4 | 336.26*  318.0 | 35  38 | 51 | 11.94 |
| AME | ESI^+^ | 273.0 | 258.14*  199.2 | 26  25 | 54 | 12.66 |

*: most abundant ion
